# Supplementary material for: Polycyclic aromatics in the Chang’E 5 lunar soils
Source: Nat Commun. 2025 Apr 16;16:3622. doi: 10.1038/s41467-025-58865-5 (PMC12003783; doi:10.1038/s41467-025-58865-5)
Supplement: Supplementary file 1 — Supplementary Information [file 41467_2025_58865_MOESM1_ESM.pdf]

S1.

Supplementary Information

Polycyclic aromatics in the Chang'E 5 lunar soils

Guangcai Zhong[1], Xin Yi[1], Shutao Gao[1], Shizhen Zhao[1], Yangzhi Mo[1], Lele Tian[1], Buqing Xu[1], Fu Wang[1], Yuhong Liao[1], Tengfei Li[1], Liangliang Wu[1], Yunpeng Wang[1], Yingjun Chen[2], Yue Xu[3], Sanyuan Zhu[1], Linbo Yu[1], Jun Li[1], Ping'an Peng[1], Gan Zhang[1, *]

[1]State Key Laboratory of Advanced Environmental Technology (SKLAET), Guangzhou Institute of Geochemistry, Chinese Academy of Sciences; Guangzhou, 510640, China.

[2]Shanghai Key Laboratory of Atmospheric Particle Pollution and Prevention (LAP3), Department of Environmental Science and Engineering, Fudan University; Shanghai, 200438, China.

[3]State Key Laboratory of Environmental Geochemistry, Institute of Geochemistry, Chinese Academy of Sciences; Guiyang, 550081, China.

***Corresponding author.** Email: zhanggan@gig.ac.cn

Supplementary Methods

Experimental materials

The glassware, stainless steel spoon, aluminum foil and basalt powder were baked at 450°C for 6 hours before use. The glass pipettes were filled with quartz wool to prevent penetration by particles from the rubber head. The quartz bottle and quartz powder were baked at 900°C for 6 hours before use. The nitrogen for evaporative blowing-down of samples was very high purity (99.999%). The inside wall of the stainless steel line for the nitrogen was successively washed with hexane, dichloromethane and methanol. After cleaning with organic solvents and drying, the nitrogen line was equipped with a hydrocarbon trap (Model BHT-2, Agilent). The perfluoroalkoxy (PFA) centrifuge tubes were successively washed with detergent, tap water and ultrapure water. They were further successively sonicated with dichloromethane, methanol and ultrapure water. The distilled ultrapure water and nitric acid were prepared with a glass distillation apparatus. The sample preparation procedures were operated in a clean laboratory (class 1000, ISO class 6 equivalent). The chemicals used in this study are summarized in Table S1.

Optical microscopy/fluorescence imaging of lunar soils

An aliquot of the lunar soil sample (14.4 mg) was weighed into a rectangular quartz bottle (size: 55 mm ×12.5 mm×2.1 mm, wall thickness: 0.8 mm) with a stainless steel spoon. The mouth of the bottle was covered with a piece of aluminum foil before a cap was screwed on. A Leica DM4 P microscope was used for observation under transmitted and reflected light. Ultraviolet fluorescence imaging used a 360 nm excitation filter in combination with a 420 nm long pass emission filter and blue fluorescence, and a 470 nm excitation filter in combination with a 515 nm long pass emission filter. In this study, no obvious evidence of organic matter was found by optical microscopy or fluorescence observation.

Analysis of lunar soils with Raman spectroscopy

The same 14.4-mg aliquot of the lunar soil sample was further analyzed by Raman spectroscopy. We used a Horiba Jobin-Yvon Lab RAM ARAMS Raman spectrometer with solid-state laser operating at a wavelength of 532 nm, coupled to an Olympus BX 41 optical microscope. A Si calibration standard was used to calibrate the spectrograph. Samples were scanned in multiple steps from 100 to 4000 cm^{-1}. Raman spectroscopy is valuable for determining the nature of any organic matter, but no obvious organic signal peak was found in this study.

Analysis of lunar soils with Py-GC-MS

Py-GC-MS determines the characteristics of pyrolysate production with respect to temperature. About 10 mg of the lunar soil sample was placed in a pyrolyzer (CDS 5200HP, CDS Analytical) and heated under a helium flow with the following temperature program: hold at 100℃ for 5 seconds; increase to 300℃ at a rate of 30℃/min; hold at 300℃ for 5 seconds; increase to 600℃ at a rate of 30℃/min; hold at 600℃ for 5 seconds; increase to 800℃ at a rate of 30℃/min. A cold trap was first held at -100°C to capture the pyrolysates during the heating stage, and was then heated to 290 °C for 3 minutes in the desorption stage.

The pyrolyzer was connected to a GC-MS (Trace GC Ultra-Trace DSQ II, Thermo Scientific) equipped with a DB-5MS fused silica capillary column (60 m × 0.25 mm i.d. × 0.25 μm film thickness). The carrier gas flow rate was 1.5 mL/min. The GC inlet was operated in splitless mode at a temperature of 290°C. The oven of the GC-MS was first held at 40 °C for 2 minutes, increased to 290°C at a rate of 3°C/min, and held at 290°C for 15 minutes. The temperature of the MS ion source was 265°C. The MS ion source was operated in electron impact mode with an electron beam energy of 70 eV. The monitoring of ion fragments was carried out in both full scan mode (*m/z* 50–650) and selected ion monitoring mode (*m/z* 67 for pyrrole, *m/z* 78 for

benzene, *m/z* 79 for pyridine, *m/z* 83 for *n*-alk-1-enes, *m/z* 85 for *n*-alkanes, *m/z* 128 for naphthalene, *m/z* 154 for biphenyl, *m/z* 167 for carbazole, *m/z* 178 for phenanthrene, *m/z* 184 for dibenzothiophene, and *m/z* 217 for benzocarbazole).

Theoretical calculation of BPCA composition for graphene sheets

For a graphene sheet with n × n array, the corresponding BPCA, which a ring converts to, is shown in Fig. S2. The number of rings which will be converted into B6CA and B5CA is calculated to be $(n-2)^2$ and 4×(n-2), respectively. The number of rings which will be converted into B4CAs is two. The number of rings which will be converted into B4CAs is also two. There are 12, 11, 10 and 9 carbon atom in B6CA, B5CA, B4CAs and B3CAs, respectively. Therefore, the contribution of individual BPCAs to the total BPCA carbon (i.e. B6CA%, B5CA%, B4CA% and B3CA%) is calculated following the equation below:

$$B6CA\% = [12 \times (n-2)^2] / [12 \times (n-2)^2 + 4\times(n-2) \times 12 + 2 \times 10 + 2 \times 9]$$

$$B5CA\% = [4\times(n-2) \times 12] / [12 \times (n-2)^2 + 4\times(n-2) \times 12 + 2 \times 10 + 2 \times 9]$$

$$B4CA\% = (2 \times 10) / [12 \times (n-2)^2 + 4\times(n-2) \times 12 + 2 \times 10 + 2 \times 9]$$

$$B3CA\% = (2 \times 9) / [12 \times (n-2)^2 + 4\times(n-2) \times 12 + 2 \times 10 + 2 \times 9]$$

According to these calculations, the BPCA compositions of graphene sheets (n = 5 to 15) were derived (Table S7).

Analysis of soluble organic compounds in the lunar soils

We obtained hot water extracts of the lunar soil during the BPCA analysis of the second batch of lunar soil samples, as mentioned above. Half of individual water extracts was used for analysis of soluble organic compounds, which was further evenly divided into three subsamples. One subsample (~1 mL) was used for analysis of amino acids and amines, one subsample for aldehydes and ketones, and the other for monocarboxylic acids. Each subsample was corresponding to ~0.17 g lunar soil.

− Amino acids and amines

The amino acids and amines were analyzed using 6-aminoquinolyl-N-hydroxysuccinimidyl-carbamate (AQC) as a derivatization reagent. Ten microliter hydrochloric acid (36-38%) was added into the subsamples in a 2-mL vial, followed by additional of 10 μL DL-norleucine ultrapure water solution (1.0 ng/μL) to check recoveries. The samples were stored at -4℃ for 4 hours, followed by freeze-drying for 12 hours. An AQC solution in acetonitrile was prepared by adding 28.5 mg of AQC into 10 mL acetonitrile. A solution A of potassium chloride and boric acid was prepared by adding 74.5 mg potassium chloride and 62.0 mg boric

acid into 10 mL ultrapure water. A solution B was prepared by adding 40.0 mg sodium hydroxide into 10 mL ultrapure water. A borate buffer solution (pH = 8.80) was prepared by adding 320μL solution A and 1000 μL solution B. After adding 70 μL borate buffer, 20 μL AQC solution and 10 μL ultrapure water into the samples dried, the mixture was shaken for well-mixing. The samples were heating at 55℃ for 10 minutes in a water bath. After cooling, the samples were diluted with 200 μL solution C, which was prepared by mixing equal volume of 1% formic acid in ultrapure water(v/v) and pure acetonitrile.

The AQC derivatization products of amino acids and amines were analyzed with the same LC-MS/MS system for BPCA analysis. The LC column was Poroshell 120, SB-C18 (2.1 mm×150 mm, 2.7 μm, Agilent). Oven temperature was 40℃. Mobile phase A was 0.1% formic acid in ultrapure water (v/v). Mobile phase B was pure methanol. Total flow rate was 0.4 mL/min. The elution gradient started with 99% A for 10 min, then from 99% to 0% A between 10-16 min, 0% A from 16 to 17 min, from 0% A to 99% A between 17-18 min, and 99% A to 23 min. The eluate containing borate buffer was discharged before 10 min and the mass spectrometer started detection afterward. The injection volume was 1 μL. The ion source of the LC-MS/MS used electrospray ionization operated in positive mode. The mass spectrometer was operated in multiple reaction monitoring (MRM) mode. The precursor ion was protonated molecular ion of the derivatization products. AQC derivates generate a characteristic product ion (m/z = 171).

External calibration curves were established with standard solutions of amino acids and amines in 0.01, 0.03, 0.06, 0.1, 0.3, 0.6, 1 and 3 ng/μL. Ten microliter of the standard solutions were added into 2-mL vials, following by addition of AQC solution and borate buffer, heating at 55℃ and dilution with solution C, in the same way as the samples. Linear calibration curves (r^2: 0.993-0.999) were used for quantification of the target compounds. The recoveries of the samples were110±24%.

- Aldehydes and ketones

The aldehydes and ketones were analyzed using the O-(2,3,4,5,6-pentafluorobenzyl) hydroxylamine (PFBHA) derivatization method[1].A solution of PFBHA was prepared by adding 2.0 mg PFBHA into 10 mL ultrapure water. One-hundred microliter PFBHA solution was added into the sample in a 4-mL vial, followed by addition of 100 ng trifluoroacetophenone (10 ng/μL in ultrapure water, 10 μL) as internal standard. The samples were stored at room temperature in the dark for 24 hours. The reaction was quenched by adding 300 μL of 0.4 M HCl solution. Dichloromethane (2 mL) was added into the solution for liquid-liquid extraction for two times. The dichloromethane layer was separated and minor water in the dichloromethane extracts was removed with anhydrous sodium sulfate, which was baked at 450℃ for 4 hours before use. The extracts

were further reduced under nitrogen blowing to ~10 μL and measured with a triple quadrupole GC-MS (GC-MS/MS, 7890A-7000A, Agilent).

The GC-MS/MS was equipped with a DB5-MS column (30 m × 250 μm × 0.25 μm). The temperature of injection port was 290℃. It worked in split mode (split ratio: 2:1). The injection volume was one microliter. The total flow rate was 1.2 mL/min. The carrier gas was helium. The oven temperature was initially hold at 60℃ for 1 min, then increased to 120℃ at 5℃/min, hold for 15 min, increased to 300℃ at 15℃/min, and hold for 5 min. The ion source was in electron impact (EI) mode. The impact energy was 70eV. The ion source temperature was 230℃. The GC and MS/MS transfer line temperature was290℃. Selected ion fragment (*m/z* = 181) was monitored for identification and quantification of target compounds.

Internal calibration curves were established with standard solutions of aldehydes and ketones. A series of standards (1, 3, 6, 10, 30, 60 and 100 ng) were added into 4-mL vials for derivatization with PFBHA, extraction with dichloromethane and measurement with GC-MS/MS, in the same way as the samples. Linear calibration curves (r^2: 0.990-0.999) were used for quantification of the target compounds.

− Monocarboxylic acids

The monocarboxylic acids were analyzed using the 2,4'-dibromoacetophenonederivatization method[2].Acidity of the samples in 2-mL vials was adjusted to pH = 8-9 with 0.05 M potassium hydroxide. The samples were desalted with a glass column (8 mm i.d., 150 cm length) packed with 4 cm cation exchange resin (Dowex 50WX8, Sigma-Aldrich). A sample was loaded into the column, followed by elution with 5 mL ultrapure water. The eluate was collected with a flask and reduced to about 0.5 mL with roti-evaporation at 50℃. The samples were transferred to 1.5-mL vials and dried under nitrogen blowing at 50℃. A solution of 2,4'-dibromoacetophenone (reagent) and dicyclohexano-18-crown-6 (catalyst) was prepared by addition of 10 μL 0.1M2,4'-dibromoacetophenone (in benzene) and 10 μL 0.01Mdicyclohexano-18-crown-6 (in acetonitrile) into 800 μL of acetonitrile. The dried samples were heated in an oven at 80℃ for 1 hour, after addition of 100 μL of this mixture. The samples were dried under nitrogen blowing and redissolved with 100 μL of hexane and dichloromethane mixture (2:1, v:v).

The target compounds were measured with the same GC-MS/MS system for analysis of aldehydes and ketones. The GC-MS/MS was equipped with a DB5-MS column (30 m × 250 μm × 0.25 μm). The temperature of injection port was 200℃. It worked in splitless mode. The injection volume was one microliter. The total flow rate was 1.5 mL/min. The carrier gas was helium. The oven temperature was initially hold at 60℃ for 2 min, then increased to 185℃ at 15℃/min, hold for 6 min, increased to 290℃

at 10℃/min, and hold for 5 min. The ion source was in electron impact (EI) mode. The impact energy was 70eV. The ion source temperature was 230℃. The GC and MS/MS transfer line temperature was 290℃. Selected ion fragment (m/z = 182.6) was monitored for identification and quantification of target compounds.

External calibration curves were established with standard solutions of monocarboxylic acids. A series of standards (1, 3, 6, 10 and 30 ng) were added into 1.5-mL vials, followed by addition of 10 μL 0.05 M potassium hydroxide. The standards were dried under nitrogen blowing at 50 ℃, and experienced derivatization and subsequent analytical procedures, in the same way as the samples. Linear calibration curves (r^2: 0.991-0.999) were used for quantification of the target compounds. Formic acid was not analyzed because derivatization of formic acid in this method was highly variable, which has been reported[2].

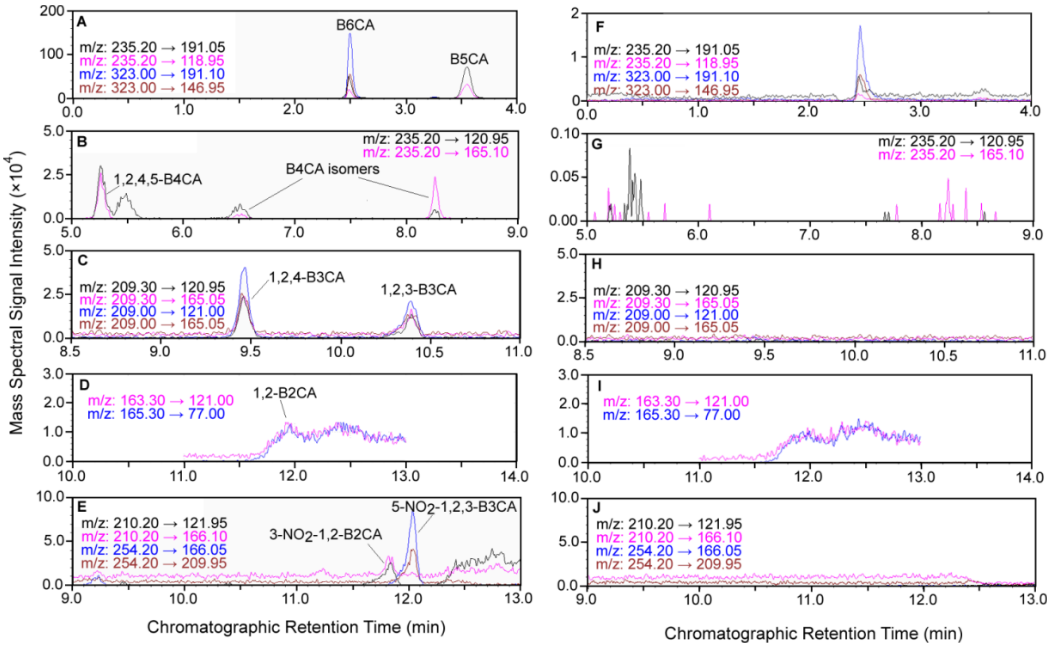

Supplementary Figure S1. A comparison of representative chromatograms of benzene polycarboxylic acids (BPCAs) extracts of the Chang'E 5 lunar soil samples (A-E) and corresponding blank samples (F-J) measured by liquid chromatography-triple quadrupole mass spectrometry (LC-MS/MS). The targeted BPCAs included mellitic acid (B6CA) and benzenepentacarboxylic acid (B5CA), pyromellitic acid (1,2,4,5-B4CA) and its two isomers, trimellitic and hemimellitic acids (1,2,4-B3CA and 1,2,3-B3CA), and 3-nitrophthalic and 5-nitro-1,2,3-benzenetricarboxylic acids (3-NO2-1,2-B2CA and 5-NO2-1,2,3-B3CA).

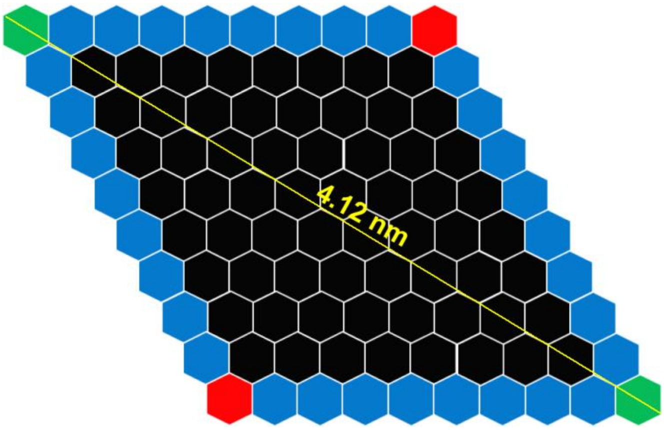

Supplementary Figure S2. Illustration of a graphene sheet with comparable condensation degree to the polycyclic aromatics of the Chang'E 5 lunar soils. The graphene sheet consists of a 10×10 benzene-ring array. Given that the C-C bond length of graphene to be 0.142 nm, diameter of the graphene sheet is calculated to be 4.12. Theoretically, the benzene rings marked in black, blue, red and green are converted into benzene polycarboxylic acids (BPCAs) substituted with six, five, four and three carboxylic groups (B6CA, B5CA, B4CA and B3CA) in BPCA method, respectively.

Supplementary Table S1. The chemicals used for benzene polycarboxylic acid (BPCA) method in this study.

Chemical	Supplier	Catalog Number
hydrofluoric acid (40%)	Guangzhou Chemical Reagent Factory	CC18-GR-0.5L
hydrochloric acid (36-38%)	Guangzhou Chemical Reagent Factory	CB11-GR-0.5L
phosphoric acid (85-90%)	ANPEL Laboratory Technologies (Shanghai) Inc.	CAEQ-4-016738-0500
disodiumhydrogen phosphate	ANPEL Laboratory Technologies (Shanghai) Inc.	CAEQ-4-012919-0250
sodium dihydrogen phosphate dihydrate	ANPEL Laboratory Technologies (Shanghai) Inc.	CAEQ-4-012929-0250
hexane	ANPEL Laboratory Technologies (Shanghai) Inc.	CBEQ-4-108708-4000
dichloromethane	ANPEL Laboratory Technologies (Shanghai) Inc.	CBEQ-4-103002-4000
formic acid	ANPEL Laboratory Technologies (Shanghai) Inc.	CAEQ-4-014784-0500
sodium hydroxide	Macklin	S817971-500g
nitric acid (65%)	Merck	84378-2.5L
sodium persulfate	Merck	71890-500G
methanol	Merck	1.06007.4008
acetonitrile	Merck	1.00030.4008

Supplementary Table S2. Parameters of liquid chromatography-triple quadrupole mass spectrometry (LC-MS/MS) for measurement of benzene polycarboxylic acids (BPCAs). Ion source of the LC-MS/MS is electrospray ionization (ESI) operated in negative mode. The mass spectrometer was operated in multiple reaction monitoring (MRM) mode.

Target Compound	Monitoring Segment (min)	Parent Ion>Product Ion (*m/z*)	Dewell Time (msec)	Collision Energy (eV)
B6CA	0.0 to 4.0	323.00>191.10(quantifier)	9	11
		323.00>146.95 (qualifier)	9	17
B5CA	0.0 to 4.0	235.20>191.05(quantifier)	9	10
		235.20>118.95 (qualifier)	9	24
B4CAs	4.0 to 15.0	253.20>120.95(quantifier)	3	15
		253.20>165.10 (qualifier)	3	10
1,2,4-B3CA	8.5 to 11.0	209.30>120.95(quantifier)	2	15
		209.30>165.05 (qualifier)	2	11
1,2,3-B3CA	8.5 to 11.0	209.00>121.00(quantifier)	2	15
		209.00>165.05 (qualifier)	2	11
3-NO_2-1,2-B2CA	4.0 to 15.0	210.20>121.95(quantifier)	2	12
		210.20>166.10 (qualifier)	2	9
5-NO_2-123-B3CA	4.0 to 15.0	254.20>166.05(quantifier)	2	14
		254.20>209.95 (qualifier)	2	9
1,2-B2CA	4.0 to 15.0	165.30>121.00(quantifier)	3	13
		165.30>77.00 (qualifier)	3	17

Supplementary Table S3. The measured and corrected $\delta^{13}C$ values of BPCA molecular markers for polycyclic aromatics in the Chang'E 5 lunar soil samples. The $\delta^{13}C$ values are expressed relative to Vienna Peedee Belemnite (VPDB). B6CA and B5CA were mellitic and benzenepentacarboxylic acids, respectively. Only the two most abundant BPCAs (B6CA and B5CA) were analyzed for the first batch of lunar soil samples CE5-B1S1, CE5-B1S2 and CE5-B1S3.The corrected values are calculated following: corrected value = measured values + offset value. The error of the offset was derived from the coefficient of variation of triplicate analyses of the offset value. The error of the corrected value was propagated from the error of the offset value.

	B6CA $\delta^{13}C$ (‰, VPDB)			B5CA $\delta^{13}C$ (‰, VPDB)		
Lunar soil Sample	measured	offset	corrected	measured	offset	corrected
CE5-B1S1	-3.30	2.96 ± 0.65	-0.34 ± 0.65	0.69	1.38 ± 0.18	2.07 ± 0.18
CE5-B1S2	-7.05	2.06 ± 0.56	-4.99 ± 0.56	3.07	0.48±1.29	3.55 ± 1.29
CE5-B1S3	-1.87	2.06 ± 0.56	0.19 ± 0.56	2.64	0.48±1.29	3.12 ± 1.29

Supplementary Table S4. Vitrinite reflectance of the type I and II kerogen samples analyzed with benzene polycarboxylic acid (BPCA) method. Calculation of the BPCA compositions is based on carbon content. BPCAs substituted with 3-6 carboxylic groups are denoted as B3CAs, B4CAs, B5CA and B6CA, respectively.

Number	Type of kerogen	Vitrinite reflectance (%)	Geological Age	Place of Production	BPCA composition (%)			
					B6CA	B5CA	B4CAs	B3CAs
#1	II	0.7	Cambrian Alum Shale Formation	Öland, Sweden	28.1	48.6	17.0	6.3
#2	I	0.7	Mesoproterozoic Xiamaling Formation	Tianjin, China	27.8	39.6	28.5	4.1
#3	II	4.0	Cambrian Maidiping Formation	Sichuan, China	55.8	18.4	22.3	3.5
#4	II	4.7	Cambrian Maidiping Formation	Sichuan, China	65.9	17.9	14.0	2.2
#5	II	not available	Ediacaran Dengying Formation	Sichuan, China	79.1	7.5	10.7	2.7
#6	II	not available	Ediacaran Dengying Formation	Sichuan, China	80.0	7.7	10.0	2.4

Supplementary Table S5. Volatile matter content of the coal samples analyzed with benzene polycarboxylic acid (BPCA) method. Calculation of the BPCA compositions is based on carbon content. BPCAs substituted with 3-6 carboxylic groups are denoted as B3CAs, B4CAs, B5CA and B6CA, respectively.

Number	Volatile matter content (%)	Rank of coal	Location of coal mines in China	BPCA composition (%)			
				B6CA	B5CA	B4CAs	B3CAs
#7	48.3	lignite	Longkou, Shandong	25.2	43.7	27.6	3.4
#8	40.1	lignite	Yima, Henan	30.8	37.7	26.6	4.9
#9	38.0	bituminous	Yanzhou, Shandong	32.3	38.3	24.9	4.5
#10	37.9	bituminous	Ningwu, Shanxi	32.6	38.4	24.7	4.2
#11	35.5	bituminous	Xuzhou, Jiangsu	40.1	34.8	20.9	4.3
#12	33.2	bituminous	Shuangyashan, Heilongjiang	36.1	37.5	22.4	3.9
#13	32.9	bituminous	Hegang, Heilongjiang	39.5	36.1	20.8	3.5
#14	32.1	bituminous	Shizhuishan, Ningxia	39.1	34.9	21.8	4.2
#15	26.9	bituminous	Xishan, Shanxi	40.9	34.6	20.5	3.9
#16	26.3	bituminous	Pingdingshan, Henan	40.3	35.3	20.4	3.9
#17	21.0	bituminous	Lishi, Shanxi	41.4	33.7	20.8	4.1
#18	19.4	bituminous	Liulin, Shanxi	40.7	34.7	20.7	3.9
#19	6.10	anthracite	Jiaozuo, Henan	53.1	28.6	16.3	2.0

Supplementary Table S6. The calculated Vitrinite reflectance (Easy%Ro) of the artificially matured type I and II kerogen samples. Calculation of the benzene polycarboxylic acid (BPCA) compositions is based on carbon content. BPCAs substituted with 3-6 carboxylic groups are denoted as B3CAs, B4CAs, B5CA and B6CA, respectively.

Artificial maturation series	Number	Heating temperature (°C)	Easy%Ro (%)	BPCA composition (%)			
				B6CA	B5CA	B4CAs	B3CAs
type I kerogen	#1	380	1.49	38.7	34.1	21.8	5.5
	#2	420	2.08	41.3	31.3	22.3	5.2
	#3	460	2.86	42.5	29.6	23.0	4.9
	#4	500	3.63	44.1	29.6	21.6	4.6
	#5	550	4.35	48.0	27.6	19.8	4.7
	#6	600	4.67	51.0	26.6	18.2	4.2
type II kerogen	#7	380	1.49	48.2	29.7	18.4	3.7
	#8	420	2.08	48.1	28.7	18.9	4.3
	#9	460	2.86	48.2	26.3	20.6	4.9
	#10	500	3.63	58.1	23.8	14.9	3.1
	#11	550	4.35	56.3	24.7	15.7	3.3
	#12	600	4.67	62.2	23.5	12.2	2.0

Supplementary Table S7. Theoretical calculation of benzene polycarboxylic acid (BPCA) compositions of graphene sheets and comparison with the polycyclic aromatics in the Chang'E 5 lunar soils. Calculation of the BPCA compositions is based on carbon content. BPCAs substituted with 3-6 carboxylic groups are denoted as B3CAs, B4CAs, B5CA and B6CA, respectively.

n×n benzene array	B6CA (%)	B5CA (%)	B4CA (%)	B3CA (%)
5×5 graphene sheet	38.8	47.5	7.2	6.5
6×6 graphene sheet	47.3	43.3	4.9	4.4
7×7 graphene sheet	53.8	39.4	3.6	3.2
8×8 graphene sheet	58.9	36.0	2.7	2.5
9×9 graphene sheet	63.0	33.0	2.1	1.9
10×10 graphene sheet	66.3	30.4	1.7	1.6
Chang'E 5 lunar soil	66.1 ± 6.3	30.1 ± 5.7	1.8 ± 0.4	2.1 ± 0.4
11×11 graphene sheet	69.1	28.2	1.4	1.3
12×12 graphene sheet	71.5	26.2	1.2	1.1
13×13 graphene sheet	73.6	24.5	1.0	0.9
14×14 graphene sheet	75.3	23.0	0.9	0.8
15×15 graphene sheet	76.9	21.7	0.8	0.7

Supplementary Table S8. Concentrations of amino acids in the Chang'E 5 lunar soil samples (ng/g).

Compound	Blank 1	Blank 2	Blank 3	Average Blank	MDL	CE5-B2S1	CE5-B2S2	CE5-B2S3
L-Arginine	1.16	1.07	1.46	1.23	1.85	2.04	< MDL	< MDL
L-Serine	0.21	0.46	1.63	0.77	3.04	< MDL	< MDL	< MDL
Glycine	0.07	0.25	0.61	0.31	1.13	3.85	2.71	3.71
L-Aspartic Acid	0.07	0.09	0.30	0.15	0.54	< MDL	< MDL	< MDL
L-Glutamic Acid	0.09	0.08	0.36	0.17	0.64	< MDL	< MDL	< MDL
L-Threonine	0.03	0.10	0.34	0.16	0.64	< MDL	< MDL	< MDL
β-Alanine	8.60	7.58	12.85	9.67	18.06	< MDL	< MDL	< MDL
L-Alanine	0.31	0.22	0.66	0.40	1.11	1.30	< MDL	< MDL
L-Proline	0.04	0.06	0.13	0.08	0.21	< MDL	< MDL	< MDL
γ-Aminobutyric acid	0.10	0.06	0.10	0.09	0.16	< MDL	< MDL	< MDL
(R)-3-Aminobutanoic acid	0.18	0.14	0.33	0.21	0.52	0.58	0.57	0.73
DL-2-Aminobutyric acid	0.06	0.07	0.14	0.09	0.22	< MDL	< MDL	< MDL
L-2-Aminobutyric acid	0.02	0.01	0.02	0.02	0.03	< MDL	< MDL	< MDL
L-Tyrosine	0.04	0.07	0.03	0.05	0.12	< MDL	< MDL	< MDL
L-Methionine	0.02	0.01	0.03	0.02	0.06	< MDL	< MDL	< MDL
2-amino-2-methyl-butanoic acid	0.14	0.24	0.67	0.35	1.20	< MDL	< MDL	< MDL
L-Valine	0.03	0.05	0.15	0.08	0.28	< MDL	< MDL	< MDL
6-Aminocaproic acid	0.03	0.03	0.06	0.04	0.09	< MDL	< MDL	< MDL
*L-Leucine and L-Isoleucine	0.02	0.06	0.15	0.08	0.28	< MDL	< MDL	< MDL
L-Phenylalanine	0.05	0.06	0.11	0.07	0.16	0.22	< MDL	< MDL

Three triplicate samples (CE5-B2S1, CE5-B2S2, CE5-B2S3,) and three procedural blanks (blank 1-3) were measured. Method detection limit (MDL) is defined as the average blank plus three times the standard deviation of the blanks. *: overlap of chromatographic peaks.

Supplementary Table S9. Concentrations of amines in the Chang'E 5 lunar soil samples (ng/g).

Compound	Blank 1	Blank 2	Blank 3	Average Blank	MDL	CE5-B2S1	CE5-B2S2	CE5-B2S3
Methylamine	0.17	0.11	0.17	0.15	0.25	15.17	15.61	17.85
Dimethylamine	0.69	0.54	0.76	0.67	1.01	1.69	1.33	1.50
Ethylamine	0.07	0.06	0.12	0.09	0.18	2.58	2.04	2.27
N-Ethylmethylamine	0.25	0.25	0.06	0.19	0.52	0.77	0.53	< MDL
Isopropylamine	0.02	0.02	0.04	0.03	0.07	0.21	0.19	0.23
Propylamine	0.03	0.02	0.05	0.03	0.07	0.19	0.17	0.21
Pyrrolidine	0.01	0.02	0.05	0.03	0.08	< MDL	< MDL	< MDL
Isobutylamine	0.04	0.01	0.12	0.06	0.22	< MDL	< MDL	< MDL
Diethylamine	4.72	3.64	4.10	4.15	5.78	< MDL	< MDL	< MDL
Butylamine	0.04	0.05	0.04	0.04	0.06	0.15	0.17	0.18
tert-Butylamine	0.02	0.02	0.01	0.02	0.02	0.07	0.06	0.09
N-Ethylpropylamine	0.18	0.17	0.24	0.20	0.31	< MDL	< MDL	< MDL
3-Aminopentane	0.19	0.18	0.25	0.21	0.32	< MDL	< MDL	< MDL
tert-Pentylamine	0.34	0.21	0.40	0.32	0.60	< MDL	< MDL	< MDL
Isopentylamine	0.06	0.04	0.06	0.05	0.09	< MDL	< MDL	< MDL
Amylamine	0.04	0.03	0.06	0.05	0.09	< MDL	< MDL	< MDL
Hexylamine	0.02	0.01	0.02	0.01	0.02	0.03	< MDL	< MDL

Three triplicate samples (CE5-B2S1, CE5-B2S2, CE5-B2S3,) and three procedural blanks (blank 1-3) were measured. Method detection limit (MDL) is defined as the average blank plus three times the standard deviation of the blanks.

Supplementary Table S10. Concentrations of monocarboxylic acids in the Chang'E 5 lunar soil samples (ng/g).

Compound	Blank 1	Blank 2	Blank 3	Average Blank	MDL	CE5-B2S1	CE5-B2S2	CE5-B2S3
Formic acid	N.A.	N.A.	N.A.	N.A.	N.A.	N.A.	N.A.	N.A.
Acetic acid	115.7	142.4	168.9	142.3	222.1	411.1	< MDL	229.7
Propionic acid	51.6	55.9	66.9	58.1	81.9	105.9	< MDL	< MDL
Isobutyric acid	6.3	5.9	6.6	6.2	7.2	9.1	< MDL	< MDL
Pivalic acid	3.7	4.4	4.5	4.2	5.6	9.3	7.1	7.1
Butyric acid	4.8	4.2	5.4	4.8	6.5	14.6	10.3	10.5
2-Methylbutyric acid	7.1	7.0	6.8	7.0	7.5	< MDL	7.8	< MDL
Isovaleric acid	7.9	7.7	7.5	7.7	8.3	< MDL	8.7	< MDL
2,2-Dimethylbutyric acid	9.7	9.7	9.7	9.7	9.8	< MDL	10.9	10.1
*3,3-Dimethylbutyric acid and Valeric acid	7.0	6.8	6.8	6.9	7.3	10.9	10.9	9.7
*2-Ethylbutyric acid and 2-Methylvaleric acid	20.0	19.7	19.1	19.6	21.0	< MDL	21.9	< MDL
*3- and 4-Methylvaleric acid	13.9	13.6	13.3	13.6	14.5	< MDL	15.4	< MDL
Hexanoic acid	0.5	0.2	0.4	0.4	0.7	2.3	1.2	1.0
Benzoic acid	4.1	1.1	3.6	3.0	7.8	28.7	17.1	17.7

Three triplicate samples (CE5-B2S1, CE5-B2S2, CE5-B2S3,) and three procedural blanks (blank 1-3) were measured. Method detection limit (MDL) is defined as the average blank plus three times the standard deviation of the blanks. *: overlap of chromatographic peaks. N.A.: not analyzed.

Supplementary Table S11. Concentrations of aldehydes in the Chang'E 5 lunar soil samples (ng/g).

Compound	Blank 1	Blank 2	Blank 3	Average Blank	MDL	CE5-B2S1	CE5-B2S2	CE5-B2S3
Formaldehyde	216.27	208.45	541.00	321.90	891.25	1233.49	< MDL	< MDL
Acetaldehyde	143.98	118.95	235.79	166.24	350.79	2194.86	< MDL	771.80
Propionaldehyde	1.95	1.34	3.94	2.41	6.49	24.30	< MDL	21.27
Isobutyraldehyde	0.57	0.74	1234.35	411.89	2548.71	< MDL	< MDL	< MDL
Trimethylacetaldehyde	1.50	1.98	8.28	3.92	15.28	< MDL	< MDL	< MDL
Butyraldehyde	12.49	5.03	17.89	11.80	31.17	< MDL	< MDL	< MDL
2-methylbutanal	2.15	2.24	12.41	5.60	23.29	< MDL	< MDL	< MDL
Isovaleraldehyde	12.15	10.07	58.00	26.74	108.02	< MDL	< MDL	< MDL
3,3-dimethylbutanal	0.04	0.04	0.36	0.14	0.70	107.84	< MDL	1.78
Pentaaldehyde	3.69	3.26	15.16	7.37	27.61	< MDL	< MDL	< MDL
2-ethylbutanal	0.23	0.12	0.72	0.36	1.31	< MDL	< MDL	4.17
Hexanal	1.12	0.89	2.55	1.52	4.22	< MDL	< MDL	7.45
Benzaldehyde	1.43	258.67	1.23	87.11	532.83	< MDL	< MDL	< MDL

Three triplicate samples (CE5-B2S1, CE5-B2S2, CE5-B2S3,) and three procedural blanks (blank 1-3) were measured. Method detection limit (MDL) is defined as the average blank plus three times the standard deviation of the blanks.

Supplementary Table S12. Concentrations of ketones in the Chang'E 5 lunar soil samples (ng/g).

Compound	Blank 1	Blank 2	Blank 3	Average Blank	MDL	CE5-B2S1	CE5-B2S2	CE5-B2S3
Acetone	17.22	14.37	61.47	31.02	110.25	< MDL	< MDL	< MDL
3-Pentone	1.87	2.22	7.25	3.78	12.80	< MDL	< MDL	< MDL
2-Pentanone	2.26	0.55	1.78	1.53	4.17	< MDL	< MDL	< MDL
3,3-Dimethyl-2-butanone	0.03	0.05	0.11	0.06	0.18	< MDL	< MDL	< MDL
Methyl isobutyl ketone	0.06	0.05	0.09	0.07	0.12	<MDL	< MDL	< MDL
2-Methyl-3-pentanone	0.01	0.01	0.02	0.01	0.03	<MDL	< MDL	< MDL
3-Hexanone	0.02	0.06	0.14	0.08	0.26	<MDL	< MDL	< MDL
2-Hexanone	0.22	0.16	0.60	0.33	1.05	<MDL	< MDL	1.54
Cyclopentanone	0.27	0.44	0.84	0.52	1.39	<MDL	< MDL	5.99
Cyclohexanone	0.21	0.20	0.44	0.29	0.69	0.71	< MDL	1.26
Acetophenone	0.13	0.14	0.29	0.19	0.45	< MDL	< MDL	< MDL

Three triplicate samples (CE5-B2S1, CE5-B2S2, CE5-B2S3,) and three procedural blanks (blank 1-3) were measured. Method detection limit (MDL) is defined as the average blank plus three times the standard deviation of the blanks.

References

1 Simkus, D. N., Aponte, J. C., Hilts, R. W., Elsila, J. E. & Herd, C. D. Compound-specific carbon isotope compositions of aldehydes and ketones in the Murchison meteorite. *Meteoritics & Planetary Science* **54**, 142-156 (2019). https://doi.org:10.1111/maps.13202

2 Nolte, C. G., Fraser, M. P. & Cass, G. R. Gas phase C2-C10 organic acids concentrations in the Los Angeles atmosphere. *Environmental Science & Technology* **33**, 540-545 (1999). https://doi.org:DOI 10.1021/es980626d
